# Happy Protest Voters: The Case of Rotterdam 1997–2009

**DOI:** 10.1007/s11205-015-0920-y

**Published:** 2015-03-14

**Authors:** Piet Ouweneel, Ruut Veenhoven

**Affiliations:** 1 Erasmus Happiness Economics Research Organization (EHERO), Erasmus University Rotterdam, Room H10-24, P.O. Box 1738, 3000 DR Rotterdam, The Netherlands; 2 Erasmus Happiness Economics Research Organisation (EHERO), Erasmus University Rotterdam, Rotterdam, The Netherlands; 3 North-West University, Potchefstroom, South Africa

**Keywords:** Political protest, Happiness, Livability of local environment

## Abstract

Protest parties are on the rise in several European countries. This development is commonly attributed to a growing dissatisfaction with life and associated with declining quality of life in modern society of the lowest social strata. This explanation is tested in a cross-sectional analysis of voting and life-satisfaction in 63 districts of the city of Rotterdam in the Netherlands, where the share of protest voters increased from 10 % in 1994 to 31 % in 2009. Contrary to this explanation protest voting appeared not to be the most frequent in the least happy districts of Rotterdam, but in the medium happy segment. Also divergent from this explanation was that average happiness in city districts is largely independent of local living conditions, but is rather a matter of personal vulnerability in terms of education, income and health. These results fit alternative explanations in terms of middle class status anxiety.

## Introduction

During the last decades a growing number of protest parties has emerged in Western Europe. This trend started with the rise of ultra right wing parties like the NPD in Germany, the Front National in France and the British National Party in England. Protest parties also figure more prominently on the left side of the political spectrum, environmentalist parties in particular. This development has been described at length (e.g., Taggart 1995; Ignazi 1996; Müller-Rommel 1998; Ignazi 2013) and has been attributed to several causes, such as the influx of immigrants (Chapin 1997; de Vos and Deurloo 1999), globalization (Hanley 2001; Leconte 2010), unemployment (Rattinger 1981; Coffé et al. 2007), retreat of the welfare state (Anderson 1996; Kriesl 1998) and the rise of meritocracy (Deegan-Krause 2007).

These societal developments are assumed to result in dissatisfaction which manifests in protest voting. In that context it is not always clear what the dissatisfaction is about precisely, about particular social issues or about life as a whole. Term such as ‘unhappy voters’ (Betz 1993), suggest that protest voting results from dissatisfaction with one’s personal life, but such suggestions are seldom substantiated empirically and the few studies that have linked protest voting to happiness did not find much difference (Veenhoven 1988; Klandermans 1989; Social and Cultural Report 2004).

In this paper we contribute to that literature with an analysis of districts in the city of Rotterdam in The Netherlands. We combined data on (1) average happiness in these districts, (2) local living conditions, (3) personal vulnerability and (4) protest voting. On that basis we seek answers to the following questions: (a) Is protest voting more frequent in the districts where average happiness is lowest? (b) If so, is that lower happiness due to poor living conditions?, or (c) Is personal vulnerability the most decisive factor behind both protest voting and unhappiness?

## Methods

Data were used from the city of Rotterdam in the Netherlands, which city is typical for West-European cities with a large working class population, a lot of migrants and a relatively low share of post-modern ‘yuppies’.

### The Case of Rotterdam

With more than 600,000 inhabitants, Rotterdam is the second largest city of the Netherlands. During the past decennia major changes have taken place in the composition and size of the population of Rotterdam. Although the city has been an immigrant town since the beginning of the industrial revolution, it was originally a white working class town. From the sixties onwards an influx of migrants from non-western nations has changed the character of the city drastically. This development was accelerated especially as the more prosperous indigenous Rotterdammers, say middle class and upper working class, began to move from the city to its surrounding satellite towns, while the have-nots, i.e. the jobless, foreign newcomers and poor pensioners remained. At present about half the population is of non-western origin.

The transition from a typical ‘dockworkers town’ to a more service and education oriented economy has also had its effects on the size and composition of the population. The typical social outline of Rotterdam today is that it is a multi-ethnic city with a relatively poorly educated population, and as a result, a high unemployment rate.

**Fig. 1:**
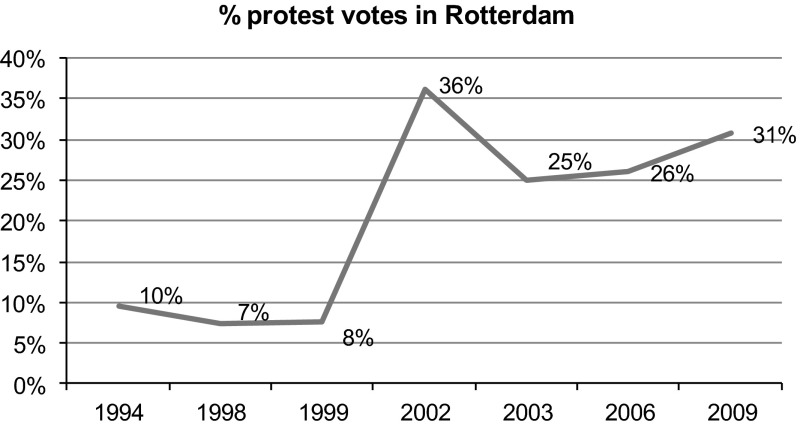
Protest voting in Rotterdam 1994–2009

Like in similar West European cities, protest voting has risen sharply in Rotterdam since the 1990s, as can be seen in Fig. 1, on which we come back later.

### Data Sources

Data were drawn from different sources: Data on happiness are taken from periodical city surveys, data on protest voting are taken from elections and data on living conditions in districts from municipal statistics.

#### Happiness

Happiness was assessed in periodical city ‘Omnibus’ surveys, which are held every 2 years among the Rotterdam population since 1997. These surveys contain a large number of questions on spare time activities, satisfaction with social, cultural and sport provisions and opportunities, as well as social, ethnical, demographic and economic indicators. They also contain a question on individual happiness.

#### Sampling

An a-select sample was drawn from all Rotterdammers aged 13–75 and an extra sample was drawn of people aged 75 and more. On one aspect the sample was not a-select: beforehand a fixed number of respondents by borough was determined to reflect the population of the respective boroughs. Within each borough the sample selection was a-select. This basic sample also consisted of nonwestern immigrants (Surinam, Antilleans, Turks, Moroccans and CapeVerdians). These groups were also interviewed in an extra face-to-face sample. This means that relatively more nonwestern immigrants were approached than without this face-to-face fieldwork. Because precisely these groups have a lower response rate the expectation was that this would have a favorable outcome for the final composition of the response. Despite that in some years an extra sample was drawn because of the disappointing response rate in those groups.

#### Response

Over the years the samples have grown to provide more robust samples and to be able to differentiate between subgroups (see Table 1). The survey has been a paper survey until 2009. In 2009 an internet survey was being held for the first time, this is probably the cause for the low response rate that year. Part of the low response rate can be accounted for by the fact that all forms that were returned empty, for example from people that have moved or have deceased, are included in the nonresponse. All in all the response rate is not bad for a city like Rotterdam. The national survey organization CBS for instance contends with a worse and more selective response in big cities.

**Table 1:** Sample and response in 7 city surveys in Rotterdam

Year	N	Response rate (%)
1997	1338	33
1999	1665	33
2001	1567	43
2003	1698	28
2005	2962	48
2007	7339	44
2009	4522	20
Total	21,091	33

When merged, these seven samples provide a dataset of 21,091 cases. We need such a big number for making a meaningful split-up of the 32 districts in Rotterdam.

#### Representivity

Representivity was achieved by comparing a number of demographic characteristics of the sample with those of the population. An extra weight factor was added in order to correct the skew distribution by borough and age to the population distribution. After weighing the sample forms a good reflection of the Rotterdam population. Although women, 45+ and natives are a little overrepresented and men, youngsters and some specific ethnic groups somewhat underrepresented. Furthermore, the response from immigrants from poor countries is somewhat lower than their population share.

#### Measure of Happiness

Happiness is the degree to which one judges positively about one’s life-as-whole. This definition is explained in more detail in Veenhoven 1984: ch. 4, who uses the term ‘happiness’ as a synonym. Thus defined, happiness is something that people have in mind and consequently it can be measured by simply asking people.

The question used in the Rotterdam city surveys reads: “Taking all things together, how happy would you say you are—very happy, happy, not too happy or not happy at all?” This question was first used in the USA (Andrews and Withey 1976) and is still common in quality of life surveys all over the world.

Validation studies have revealed that the answers to such questions on happiness produce valid outcomes. People understand what the question is about and respond accordingly. The rate of ‘don’t know’ answers is typically less than 1 % (Veenhoven 1984, ch 3). Yet reliability is not too good, since the difference between ‘very happy’ and ‘happy’ is not easy to see and because responses can be tilted by things such as the place of the item in the questionnaire and the weather in de day of the interview (see Veenhoven 1984). Such random variations balance out in big samples, so reliability is not a problem in this study.

#### Transformation of Scores to Scale 0–10

For ease of presentation we transformed the scores on this 4-point scale into 0 tom 10 school marks, using the following numerical equivalents for verbal response options as estimated by Veenhoven (1993):

- Very happy 9.3
- Happy 7.0
- Not too happy 4.0
- Not happy at all 1.0

#### Protest Voting

Protest voting in an election demonstrates the caster’s dissatisfaction with main stream candidates. Protest voting does not necessarily take the form of a valid vote (voice) but can also take the form of abstention from voting (exit). Voiced protest is typically a vote in favor of a minority or fringe candidate, either from the far left, far right or self-presenting of a candidate foreign to the political system (Wikipedia 2014). Protest is also voiced by ‘white’ votes.

In this analysis we measure protest voting both by the percentage of protest votes of all valid votes and by the percentage of white votes and nonvoters combined. In the analysis one should bear in mind that the number of white votes is very small in all districts and don’t even figure marginally compared to the number of nonvoters.

The years of which the election results were analyzed covers the period 1998 to 2009. In this period all elections held were included: community elections, provincial elections as well as national elections. The number and names of the protest parties differed between elections but also between years in which the elections were held. Some parties that participated in 1998 have disappeared in 2009, while others still did not exist in 1998. The following parties are regarded as ‘protest party’: NVU, CP’86, CD, SP, Stadspartij, Leefbaar, LijstFortuijn, TrotsOpNederland, LPF and PVV. Attracting most voters in comparison to the other protest parties the PVV (Party for Freedom) presents itself as the party for the ‘common man’ opposed to the political elite. It is furthermore eurosceptic, anti-islam and anti-immigrant like the other Dutch populist parties that operate in the margin, with the exception of the SP (Socialist Party) that operates on the left side of the political spectrum.

The main message of these parties is that they are against the political establishment, such as nicely illustrated by the poster of the SP party, on which a tomato is thrown. Much of the protest focuses on the growing number of migrants in the city.

All protest votes were summed and a percentage was calculated on the base of the total number of valid votes. In 1998 and 1999 the share of protest votes was not very large, but the share rose to 36.1 % in 2002. This means that 1 out of every 3 votes was on a protest party.

White voters and nonvoters were computed as a percentage of the total number of eligible votes, overall this figure was 38 %. Data on voting is available for 63 districts of Rotterdam. The city counts more districts but districts with less than 75 inhabitants like industrial sites were left out. The election results were incorporated in the main database.

#### Further Characteristics of Districts

For policy reasons every year since 2007 a ‘Social Index Score’ is computed, which consists of objective and subjective indicators about the following characteristics of districts:

Vulnerability of inhabitants:

- proficiency of the Dutch language
- income level
- health
- education level

Social cohesion:

- self perceived social cohesion in the neighborhood
- rate of removals in the neighborhood.

Social participation:

- involvement in work and school
- social contacts
- socio-cultural activities
- social commitment.

Living environment:

- appropriate housing
- adequate provisions
- absence of discrimination
- no pollution and nuisance.

## Analysis

Having set the scene, we can now answer the research questions mentioned in Sect. 1.

### More Protest Votes in Unhappy Districts*?*

The first question was whether protest voting is a sign of general dissatisfaction with one’s own life. If so, a ‘happy’ district would count few protest votes. At first sight, the data rather show the reverse, protest voting being more common in the happier districts. r = +.34 (*p* < .01).

Yet this simple correlation can be misleading, since the votes of migrants may distort the picture. Remember that about half of the population of Rotterdam consists of immigrants with a lower social status, who are unlikely to vote for anti-migrant protest parties.

The zero-order correlation between the % of immigrants and the rate of protest votes in a district confirmed this: r = −.62 (*p* < .001). The correlation between happiness and nonvoters was similar with r = −.65 (*p* < .001).

If the percentage of immigrants in a district is held constant the partial correlation resulted in the expected direction: rpc = −.31 (*p* < .05), meaning that there is a modest but significant relation between happiness and protest voting. The relation with the percentage of nonvoters is even stronger with r = −.40 (*p* < .001). This pattern appears more clearly in the split-up in 3 equal groups presented in Table 2, which shows that the most and least happy districts have the lowest percentage of protest votes, while in the middle ‘happy’ category more than 27 % of voters vote on a protest party. An explaining factor might be that the growth of nonwestern immigrants in this middle group was highest of all three groups with 33.3 % between 1997 and 2009.

**Table 2:** Happiness, protest voting and immigrants in 63 districts in city of Rotterdam

Mean happiness	% Protest votes	% White and nonvoters	% Immigrants
7.6	22.9	30.3	15.9
7.2	27.4	37.0	22.5
6.8	19.2	46.3	61.4

The relation is linear between happiness and white and non-voters: the unhappiest districts have the highest percentage of nonvoters, followed by the middle and happiest groups respectively. The reasons for not voting may not only be protest voting but also due to lack of political commitment and limited understanding of the Dutch language.

**Fig. 2:**
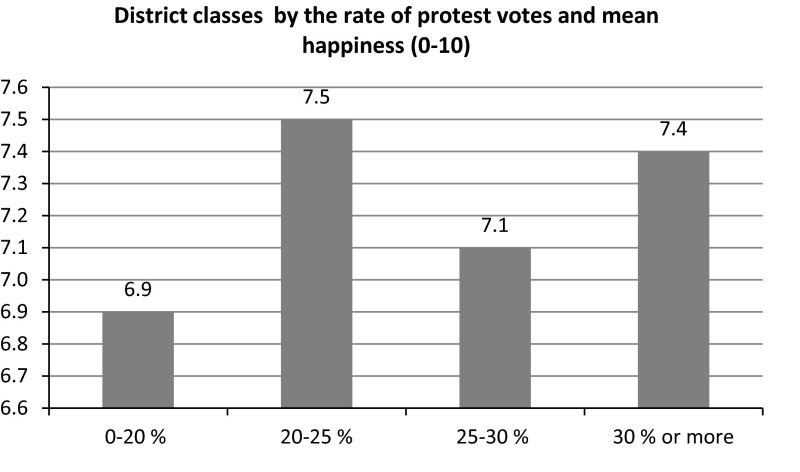
Average happiness and protest voting in 63 districts in city of Rotterdam

When we split the districts by the percentage of protest votes (white and non-votes excluded), a similar result is found, see Fig. 2. In the group of districts with the lowest percentage of protest votes, i.e. under 20 %, people are with an average of 6.9 least happy. But the group which follows, with 20–25 % of protest votes counts the districts with the happiest inhabitants with an average happiness score of 7.5. But the people in the districts with the highest percentage of protest votes, more than 30 %, are almost as happy with 7.4.

**Fig. 3:**
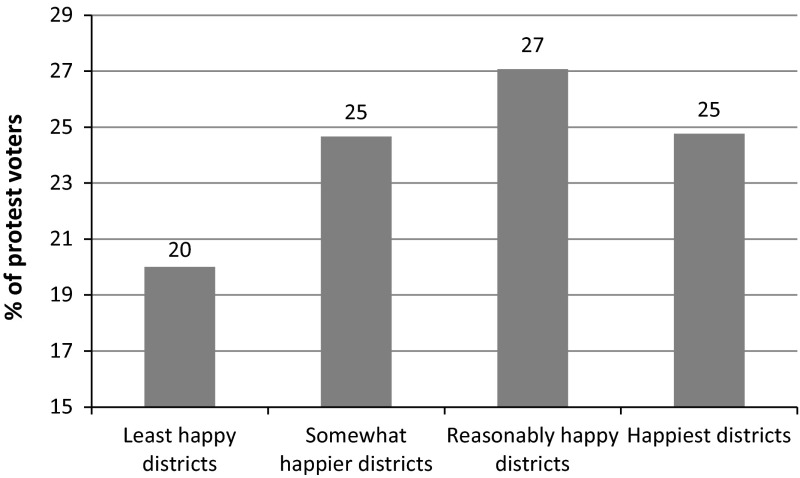
Happiness in districts by the % of protest votes

We can also approach the relation between happiness and protest votes by splitting the districts in 4 happiness levels (see Fig. 3). We see a similar nonlinear pattern: the least happy districts have the lowest percentage of protest votes (20 %), while the middle group of ‘reasonably happy’ districts are characterized by the highest percentage of protest voters (27 %) and in the happiest districts 25 % of the voters vote on a protest party. Because the correlation between  % of protest votes and  % of non-western immigrants is strongly negative (r = −0.75, *p* < .01) we repeated the analysis by controlling the  % of nonwestern immigrants. Than the pattern is in the expected direction, meaning that the least happy districts count the highest percentage of protest votes (r = −0.44, *p* < .01).

Why this non-linear pattern? Close inspection of the Tables 3, 4 reveals that moderate happiness tends to go with protest voting in two kinds of districts.

**Table 3:** Percentage of protest voters by district

Buurt	% Protest	Buurt	% Protest	Buurt	% Protest
Spangen	12.8	Struisenburg	22.0	Pernis	27.0
Afrikaanderwijk	13.7	Blijdorp	22.1	Prinsenland	27.3
Feijenoord	13.7	Tarwewijk	22.4	Zevenkamp	27.5
Bospolder	15.3	Schiebroek	22.5	Nieuw Crooswijk	27.6
Tussendijken	15.4	Liskwartier	22.7	Lombardijen	27.7
Hillesluis	15.8	Cool	23.5	Hoogvliet-Zuid	28.1
Schiemond	16.1	Hillegersberg-Noord	23.5	Oud-Mathenesse	28.3
Nieuwe Westen	16.6	Stadsdriehoek	23.8	Zuidplein	28.4
Oude Westen	16.8	Bergpolder	24.4	Carnisse	28.6
Middelland	19.1	Klein Polder	24.7	Ommoord	28.6
Nieuwe Werk	19.5	Hillegersberg-Zuid	24.8	Oosterflank	28.8
Molenlaankwartier	20.1	s-Gravenland	24.8	Zestienhoven	28.9
Kralingen-Oost	20.2	Pendrecht	25.0	Overschie	29.0
Kralingen-West	20.4	Het Lage Land	25.0	Beverwaard	29.2
Delfshaven	20.7	Terbregge	25.1	Groot IJsselmonde	29.3
Kop van Zuid-Entrepot	20.7	Noordereiland	25.4	Zuidwijk	29.6
Provenierswijk	20.8	Hoogvliet-Noord	25.6	Nesselande	31.4
Agniesebuurt	20.9	Rubroek	25.7	Oud IJsselmonde	32.1
Oud Crooswijk	21.6	Kralingesveer	26.6	Wielewaal	33.3
Hoek van Holland	21.8	Katendrecht	26.6	Landzicht	34.6
Oude Noorden	21.9	Oud-Charlois	26.6	Vreewijk	35.7
Bloemhof	21.9	De Esch	26.8	Heijplaat	36.1
				Rotterdam	18.3

**Table 4:** Mean happiness in 63 districts of Rotterdam

Neighborhood	Mean	Neighborhood	Mean	Neighborhood	Mean
Nesselande	8.0	Wielewaal	7.2	Rubroek	7.0
Kralingen Oost	7.7	Hillegersberg Noord	7.2	Oud-Mathenesse	7.0
Terbregge	7.6	Groot Usselmonde	7.2	Vreewijk	7.0
Molenlaankwartier	7.5	de Esch	7.2	Kleinpolder	7.0
Blijdorp	7.5	Zevenkamp	7.2	Nieuwe Westen	7.0
Struisenburg	7.4	Schiebroek	7.2	Oud-Charlois	6.9
Hoek v. Holland	7.4	Proven ierswijk	7.1	Oude Noorden	6.9
s-Graven land	7.4	Zuidplein	7.1	Oude Westen	6.9
Overschie	7.4	KopvZuidEntrepot	7.1	Carnisse	6.9
Oud Usselmonde	7.4	Lombardijen	7.1	Tarwewijk	6.9
Pernis	7.3	Bergpolder	7.1	Afrikaanderwijk	6.9
Kralingseveer	7.3	Cool	7.1	Bloemhof	6.9
Stadsdriehoek	7.3	Kralingen West	7.1	Spangen	6.9
Hillegersberg Zuid	7.3	Hilles lu is	7.1	Schiemond	6.9
HoogvlietZuid	7.3	Zuidwijk	7.1	Delfs haven	6.9
Ommoord	7.3	Beverwaard	7.1	Pendrecht	6.9
Heijplaat	7.2	Oosterflank	7.1	Nieuw Crooswijk	6.8
Hoogvliet Noord	7.2	Agniesebuurt	7.1	Feijenoord	6.8
Het Lage Land	7.2	Middelland	7.0	Bos polder	6.8
Liskwartier	7.2	Noordereiland	7.0	Tussendijken	6.7
Prinsenland	7.2	Katendrecht	7.0	Oud Crooswijk	6.7
				Rotterdam	7.1

### Village-Like Districts

Districts of Rotterdam with a village structure score high on the rankings of protest votes. The combination of a more closed white community, a safe haven in the big city, with a vulnerability for immigration of poor immigrants explains why people in these districts vote on right wing protest parties that promise to keep immigrants out. Often these districts lie isolated from the rest of Rotterdam, but not always. Examples of these ‘villages’ with the percentage of protest votes between parentheses are Kralingseveer (26.6 %), Pernis (27.0 %), Oud-IJsselmonde (32.1 %), Overschie (29.0 %), Wielewaal (33.3 %) and Vreewijk (35.7 %).

Comparing happiness levels with nonvoters the pattern is again linear, because nonvoters consist also of groups without commitment to politics and groups with not enough mastery of the Dutch language.

### Threatened Lower Middle-Class Districts

The somewhat more prosperous districts of the lower middle-class are mainly situated in the outer ring of Rotterdam. The housing distribution of these districts is characterized by a mix of owner occupied houses and social housing projects. Because of the state subsidies low income groups have access to this latter category even to the somewhat roomier terraced houses. The consequence is that these originally ‘white’ districts experience a growing inward flow of nonwestern immigrants. The growth of nonwestern immigrants has been the highest compared to other districts. Different cultural norms, unemployment and hence a lack of integration collide with native norms and attitudes, which seems to result in protest voting of the native population.

### Unhappiness in Districts a Matter of Livability or Life-Ability?

The theory that protest voting comes from unhappiness because of poor living conditions presumes a strong effect of local living conditions on happiness. Yet another theory holds that bad districts attract vulnerable people, who do not cope well with life anyway and would have been equally unhappy in better neighborhoods. Which of these theories fits our data best?

Vulnerability of inhabitants stands out as the strongest predictor of average happiness of the four characteristics mentioned in Sect. 2.2. See Table 5. At first sight the zero-order correlations of each of the district characteristics with happiness are firm. But when controlled for vulnerability of inhabitants the partial correlations come close to zero and are all not significant.

**Table 5:** Correlations of district characteristics with happiness

Characteristics of districts	Zero-order correlation with average happiness	Vulnerability of inhabitants partialled out
Environment	+.74*	+.04
Social cohesion	+.55*	+.02
Social participation	+.69*	−.14
Vulnerability of inhabitants	+.91*	

* *p* < .01

One could argue that because of multicollinearity between these variables the zero-order correlation of vulnerability with happiness (r = +.91, *p* < .01) will also drop to almost zero when controlled for the other district characteristics. This however is not the case: When controlled for environment, social cohesion and social participation the partial correlation between happiness and vulnerability of inhabitants is still firm and significant with an rpc = +.45 (*p* < .01). So also from this perspective, the path from local livability → happiness → protest voting is small.

## Discussion

### Explanations

In discussions on societal discontent a common argument is that the have-nots show their discontentment with the red pencil, i.e., by protest voting. Yet it is not the underclass of modern ‘paupers’ that vote on protest parties, but rather the lower middle class and upper working class of ‘established’ people who feel threatened in their modest prosperity and life style by nonwestern newcomers that came to live in previously white districts with a mixture of privately owned houses and social housing projects. These natives are confronted with other cultures and diminishing social cohesion, which leads to feelings of alienation. Durkheim’ s anomia theory comes closer in explaining voting behavior. This explanation fits the wider theory of ‘threatened middle class’ (e.g., Littrell et al. 2010).

### Limitations

This analysis was done on the level of districts rather than on an individual level, because data on voting and livability are only available at the district level. A multi-level analysis is therefore not possible. Still these district level data are richer than could have been obtained with individual level survey data only.

In cross-sectional studies like this there is always the ghost of collinearity on the verge. When we look for instance at the components of personal capacities: mastery of the Dutch language, net family income, subjective health and education level, there is collinearity with the  % of nonwestern immigrants, with well to do and poor neighborhoods and even with health.

The focus of this research was on local government and happiness. This means that the outcome of this study cannot be generalized to a national level. Local conditions are not the same as national political topics.

## Conclusion

Protest voting cannot be attributed to general dissatisfaction with life in modern society, but draws on more specific problems over which local policy makers have some control.

## Appendix

### Aspects of livability in 63 districts in city of Rotterdam

All rated on scale 0–10

**Table:**

District	Social index score	Capacities	Sufficient education	Good health	Sufficient income	Mastering the language	Living environment	No discrimination	Good housing	Other provisions
Afrikaanderw ijk	4.9	3.6	4.6	4.1	2.8	2.9	5.1	5.1	4.2	5.9
Agniesebuurt	5.5	4.9	6.5	5.4	3.9	3.8	5.7	5.9	5.4	5.5
Bergpolder	6.2	6.5	7.5	6.6	5.9	6.0	6.4	7.1	5.8	5.7
Beverw aard	5.7	5.1	5.4	4.9	5.0	4.9	5.9	5.4	6.5	5.6
Blijdorp	7.4	8.3	7.5	7.8	8.9	8.9	7.6	8.0	8.0	6.4
Bloemhof	4.8	3.9	4.5	4.5	3.1	3.6	5.1	5.6	4.4	5.5
Bos polder	5.2	4.0	5.3	4.0	3.3	3.4	5.3	5.9	4.5	5.7
Carnisse	5.1	5.2	6.0	4.7	5.6	4.3	5.3	5.0	5.0	6.0
Cool/Nieuw e Werk/Dijkzigt	6.5	6.6	8.2	6.2	6.4	5.8	6.6	6.9	7.4	5.8
De Esch	6.4	6.6	7.3	5.4	6.5	7.0	6.9	6.7	7.5	6.0
Delfs haven	5.4	4.8	7.0	4.7	3.4	4.2	5.5	5.9	5.2	5.6
Feijenoord	4.8	3.7	4.5	4.4	2.8	3.2	5.1	5.6	4.2	5.4
Groot Usselmonde	6.4	6.0	5.8	5.5	5.7	7.0	6.5	6.2	7.1	6.2
Heijplaat	6.2	5.4	5.0	5.1	5.2	6.2	6.5	6.7	7.0	5.2
Het Lage Land	6.9	7.2	7.2	6.6	7.5	7.3	7.6	7.6	7.7	7.1
Hillegersberg-noord	7.3	7.3	6.8	6.6	7.4	8.3	7.7	7.6	7.9	6.8
Hillegersberg-zuid	7.5	8.1	7.6	7.8	8.6	8.4	7.3	7.6	7.3	6.1
Hilles luis	5.0	4.0	5.1	4.4	3.4	3.1	5.1	5.8	4.4	5.4
Hoekvan Holland	8.1	7.7	7.4	6.1	8.3	8.9	8.5	8.7	8.5	7.7
Hoogvliet-noord	6.3	5.7	6.1	4.7	6.6	5.4	6.7	6.1	7.5	6.2
Hoogvliet-zuid	7.2	7.1	7.3	5.9	7.3	8.1	7.2	7.3	8.0	6.5
Katendrecht	5.6	4.6	5.5	4.6	3.7	4.7	6.0	5.6	5.9	5.8
Kleinpolder	5.5	4.9	5.2	5.0	4.3	5.4	5.7	5.1	5.6	5.8
Kop van Zuid-Entrepot	6.4	6.4	6.6	6.7	6.9	5.5	6.4	6.5	6.5	5.5
Kralingen Oost/Kralingse Bos	7.6	8.3	8.2	8.2	9.0	7.7	7.2	6.7	8.1	6.0
Kralingen-west	6.0	5.4	7.3	4.6	4.5	5.4	6.2	6.4	5.9	6.2
Kralingseveer	7.8	8.0	7.0	8.2	8.8	8.2	7.5	8.4	7.1	6.4
Liskw artier	6.4	5.9	7.2	5.8	5.0	5.5	6.4	6.2	6.2	6.0
Lombardijen	5.8	4.8	5.2	4.4	4.8	4.9	6.6	6.7	6.5	6.4
Middelland	5.7	5.1	5.8	5.5	4.4	4.7	5.9	6.5	6.2	5.7
Molenlaankw artier	8.1	8.6	8.0	8.3	9.2	8.9	8.1	8.1	8.7	6.6
Nesselande	7.7	8.4	7.4	7.9	9.3	8.9	7.6	7.4	8.3	5.8
Nieuw Croosw ijk	5.3	4.5	5.8	4.7	3.5	4.2	5.4	5.5	5.3	5.8
Nieuw e Westen	5.5	4.8	6.3	5.4	3.8	3.8	5.6	7.0	4.9	5.6
Noordereiland	6.1	5.5	5.6	6.1	4.7	5.7	6.5	6.9	6.4	5.3
Ommoord	7.1	7.3	7.2	6.3	7.2	8.7	7.1	6.6	8.1	6.9
Oosterflank	6.5	6.1	6.7	4.9	6.9	5.8	6.8	7.0	7.2	6.4
Oud Croosw ijk	5.1	4.1	5.4	3.7	3.3	3.9	5.5	5.8	5.4	5.9
Oud Usselmonde	6.9	7.2	6.6	6.7	8.2	7.5	7.0	6.6	7.3	5.5
Oud/Nieuw Mathenesse/Witte Dorp	5.2	4.7	5.6	4.8	4.5	3.9	5.8	6.3	5.3	5.2
Oud-Charlois	5.1	4.4	5.4	4.1	4.0	4.1	5.4	5.0	5.2	5.9
Oude Noorden	5.3	4.5	5.5	4.7	3.3	4.5	5.7	6.3	4.9	5.8
Oude Westen	5.5	4.1	5.6	3.9	3.2	3.7	5.5	5.8	5.1	5.8
Overschie e.o.	7.1	7.4	6.7	6.8	7.8	8.5	6.7	6.7	6.3	5.8
Fendrecht	4.9	4.1	5.1	4.0	3.6	4.0	5.4	5.2	5.5	5.8
Fernis	7.5	7.7	6.6	6.5	8.5	9.3	7.4	7.4	7.3	6.7
Frinsenland	7.2	7.2	7.4	5.7	7.9	7.6	7.4	6.9	8.0	6.6
Proven iersw ijk	6.0	5.7	6.8	6.0	4.8	5.4	6.3	6.4	5.9	6.3
Rubroek	6.0	5.8	7.0	5.0	5.0	6.2	6.2	6.4	7.0	6.0
s-Gravenland	7.9	8.5	7.8	8.1	9.3	8.8	7.7	8.0	8.5	5.9
Schiebroek	6.8	6.5	6.3	5.9	6.4	7.3	6.9	6.3	7.4	6.7
Schiemond	5.6	4.6	5.6	4.3	3.8	4.4	6.3	6.8	5.8	5.4
Spangen	5.3	4.3	5.2	4.6	3.7	3.6	5.3	5.5	4.4	5.9
Stadscentrumtotaal	6.3	6.3	7.8	6.2	6.4	4.8	6.4	6.7	7.2	5.8
Stadsdriehoek/C.S. Kw artier	6.8	7.6	8.8	7.3	8.8	5.6	7.0	7.0	8.6	5.9
Struisenburg	6.8	7.3	7.4	7.3	7.8	6.9	6.9	7.3	8.0	5.3
Tarwewijk	4.8	4.1	5.0	4.1	3.5	3.8	5.1	5.6	4.8	5.2
Terbregge	7.8	8.1	7.2	8.5	8.8	8.1	7.5	8.3	7.1	5.7
Tussendijken	5.1	3.8	5.1	4.0	3.0	3.1	5.6	6.0	4.8	5.9
Vreew ijk	6.1	5.7	5.9	4.7	4.5	7.7	6.6	6.7	6.4	6.0
Wielewaal	6.5	5.4	4.5	5.4	3.8	8.0	7.7	7.3	8.0	6.6
Zevenkamp	7.0	7.0	6.5	6.1	7.5	8.0	6.9	7.2	7.0	6.4
Zuidw ijk	5.7	4.4	5.4	4.0	3.7	4.6	6.5	6.1	6.2	6.7
Rotterdam	6.0	5.4	6.2	5.2	4.9	5.2	6.3	6.5	6.3	5.9

District	No pollution and social nuisance	Participation	Work and school	Social contacts	Social and cultural activities	Social commitment	Social cohesion	Moving mutations	Self-experienced cohesion of neighborhood
Afrikaanderw ijk	5.1	5.2	4.0	5.8	5.1	5.9	5.7	6.3	5.2
Agniesebuurt	6.2	5.6	5.1	5.6	6.5	5.3	5.7	5.9	5.4
Bergpolder	7.1	6.6	5.3	6.4	8.1	6.4	5.2	4.6	5.9
Beverw aard	6.0	5.9	6.1	6.0	5.8	6.0	5.7	6.7	4.8
Blijdorp	8.1	7.0	6.6	6.7	7.9	6.8	6.5	5.6	7.5
Bloemhof	5.0	5.1	4.2	5.2	5.0	6.1	5.2	5.5	4.9
Bos polder	5.1	5.8	5.0	5.7	6.0	6.5	5.9	5.9	5.8
Carnisse	5.1	5.6	5.0	5.5	6.3	5.8	4.4	4.5	4.2
Cool/Nieuw e Werk/Dijkzigt	6.4	6.7	5.3	6.9	8.4	6.1	5.9	5.1	6.6
De Esch	7.3	6.5	6.2	5.9	7.3	6.6	5.5	5.7	5.4
Delfs haven	5.5	6.2	5.0	6.1	7.7	5.8	5.2	5.0	5.4
Feijenoord	5.2	4.7	4.5	5.0	3.9	5.3	5.5	6.2	4.9
Groot Usselmonde	6.7	6.1	6.2	5.9	5.6	7.0	6.7	7.1	6.3
Heijplaat	7.0	6.5	6.3	6.8	5.8	7.1	6.5	6.6	6.3
Het Lage Land	7.9	6.3	5.8	5.9	6.4	7.2	6.6	6.1	7.2
Hillegersberg-noord	8.4	7.1	7.2	6.4	7.6	7.3	7.3	6.2	8.4
Hillegersberg-zuid	8.0	7.3	7.0	7.0	7.5	7.6	7.2	6.2	8.1
Hilles luis	4.7	5.5	5.2	5.1	5.6	6.0	5.6	5.6	5.5
Hoekvan Holland	9.1	7.6	7.9	8.6	6.6	7.4	8.5	8.4	8.5
Hoogvliet-noord	6.9	6.4	7.2	5.6	6.1	6.7	6.5	7.2	5.9
Hoogvliet-zuid	7.3	6.7	7.6	6.0	6.3	6.8	7.7	7.8	7.5
Katendrecht	6.5	5.7	4.9	5.4	6.7	6.0	6.2	5.9	6.5
Kleinpolder	6.4	5.5	5.5	5.3	5.4	5.8	5.8	6.2	5.3
Kop van Zuid-Entrepot	7.2	6.6	6.3	5.5	7.4	7.1	6.0	6.2	5.8
Kralingen Oost/Kralingse Bos	8.0	7.9	6.5	8.2	9.1	7.7	7.0	5.6	8.4
Kralingen-west	6.4	6.4	5.4	6.5	7.4	6.2	5.9	5.4	6.4
Kralingseveer	8.1	7.1	8.2	6.5	6.5	7.0	8.5	8.6	8.4
Liskw artier	7.4	6.6	6.4	5.8	7.4	6.8	6.5	5.8	7.2
Lombardijen	6.7	5.7	5.3	5.1	5.7	6.8	6.1	6.2	6.1
Middelland	5.3	6.5	5.3	7.0	7.8	5.7	5.2	5.0	5.5
Molenlaankw artier	9.0	7.8	9.3	6.8	7.4	7.6	7.8	7.1	8.4
Nesselande	8.8	7.5	8.5	7.0	7.3	7.0	7.3	6.9	7.7
Nieuw Croosw ijk	5.1	6.0	4.2	6.4	7.2	6.3	5.1	5.5	4.7
Nieuw e Westen	5.1	6.0	5.3	5.7	6.8	6.2	5.6	5.6	5.7
Noordereiland	7.6	6.2	5.0	5.5	7.6	6.9	6.3	6.6	5.9
Ommoord	6.7	6.5	7.3	5.2	6.2	7.4	7.5	7.8	7.3
Oosterflank	6.7	6.2	6.6	5.7	5.9	6.6	6.9	7.4	6.5
Oud Croosw ijk	4.9	5.4	4.6	5.4	6.1	5.6	5.5	6.2	4.8
Oud Usselmonde	8.5	6.5	7.0	6.1	6.4	6.7	6.9	7.0	6.8
Oud/Nieuw Mathenesse/Witte Dorp	6.2	5.8	5.3	5.3	6.4	6.1	4.7	4.8	4.6
Oud-Charlois	5.4	5.8	5.7	5.1	5.9	6.3	4.9	5.4	4.5
Oude Noorden	5.9	5.5	5.0	5.8	5.8	5.5	5.6	5.8	5.5
Oude Westen	5.1	6.3	4.8	6.6	7.0	6.8	6.2	5.8	6.5
Overschie e.o.	7.9	6.8	6.6	6.4	6.7	7.7	7.5	7.7	7.4
Fendrecht	5.0	5.4	4.8	4.9	5.3	6.5	4.9	5.5	4.3
Fernis	8.3	7.0	8.1	6.2	5.7	7.9	8.0	7.9	8.2
Frinsenland	8.3	6.9	7.7	6.2	7.0	6.7	7.4	7.4	7.5
Proven iersw ijk	6.6	6.4	5.3	5.9	8.0	6.5	5.6	5.5	5.7
Rubroek	5.6	6.5	5.9	6.5	7.4	6.0	5.6	5.7	5.5
s-Gravenland	8.5	7.6	9.1	6.2	7.6	7.6	7.6	7.3	7.8
Schiebroek	7.2	6.6	6.8	5.7	6.6	7.4	7.2	6.6	7.9
Schiemond	7.1	5.8	5.8	5.1	6.7	5.7	5.9	6.1	5.7
Spangen	5.4	5.8	5.1	5.3	6.3	6.3	5.8	5.8	5.9
Stadscentrumtotaal	5.9	6.8	5.5	7.0	8.1	6.4	5.8	5.1	6.5
Stadsdriehoek/C.S. Kw artier	6.3	6.9	5.5	7.4	8.4	6.2	5.7	4.8	6.5
Struisenburg	6.9	7.2	5.5	8.2	8.9	6.1	5.9	4.8	7.0
Tarwewijk	4.8	5.6	4.2	5.6	6.3	6.3	4.3	4.3	4.3
Terbregge	8.9	7.4	8.2	5.9	7.8	7.8	8.1	8.4	7.7
Tussendijken	5.8	5.0	4.5	4.7	5.2	5.5	5.8	5.3	6.3
Vreew ijk	7.2	5.3	5.1	5.5	4.2	6.4	6.8	7.4	6.3
Wielewaal	8.7	5.4	3.5	5.8	5.0	7.1	7.6	8.2	7.1
Zevenkamp	6.9	7.1	7.0	7.3	6.7	7.3	7.2	7.3	7.0
Zuidw ijk	7.1	5.3	4.3	5.2	5.6	6.2	6.4	6.1	6.6
Rotterdam	6.5	6.3	6.0	5.8	6.6	6.7	6.1	5.9	6.4
